# A new approach to improving traffic literacy and social capital for behavioral modification, using Advocacy, Social marketing, Campaigns and Social mobilization techniques, with TOT strategy (SHEPMODEL)

**Published:** 2019-08

**Authors:** Gholamreza Nourabadi, Mohammad Mehdi Gouya, Mohammad Hossein Somi, Ahmadreza Jodati, Morteza Ghojazadeh, Farah Shakibfar, Mehran Rahimi

**Affiliations:** ^*a*^Liver and Gastrointestinal Diseases Research Center, Tabriz University of Medical Sciences, Tabriz, Iran.; ^*b*^Center for Disease Control, Ministry of Health and Medical Education, Tehran, Iran.; ^*c*^Cardiovascular Research Center, Tabriz University of Medical Sciences, Tabriz, Iran.; ^*d*^Research Center for Evidence Based Medicine (RCEBM), Tabriz University of Medical Sciences, Tabriz, Iran.

## Abstract

**Background:**

The weakness of the health sector in providing homogeneous and suitable educational content for public and lack of a systematic approach to implementing programs at the national level to province and county levels with the aim of modifying the individual and Traffic related behaviors. Local setting: In Iran, the Ministry of Health and Medical Education (MOH) is responsible for maintaining and improving public health status. Transferring health knowledge to people is one of the duties of the health staff in all occupational and professional branches.

**Methods:**

This educational model has three phases: assessment, implementation and evaluation. Related changes: In this study, which is one of the first surveys of its kind, 470 MOH staff and NGOs representatives from different provinces participated in training workshops based on the Systematic Comprehensive Health Education and Promotion model (SHEPmodel) traffic accidents, and evaluated the effectiveness and satisfaction level of the model.

**Results:**

One of the most important lessons learned from the educational programs of this model is the sense of satisfaction the trainers gained from the translated scientific health contents into the intelligible public language along with the existence of a systematic implementation structure. Furthermore, reports and studies conducted over more than a decade of implementation of programs are indicative of the empowerment of trainers with four techniques and seven educational and communicational skills to use in public health interventions for modifying public health behavior.

**Conclusions:**

The results of the present study showed that the effectiveness of SHEPmodel was higher than the traditional methods; so that 86% of the participants attending the trainer workshop evaluated this model as highly effective.

**Keywords:**

Traffic literacy, Health literacy, Training of trainers, Cascading format, Social capital, Advocacy

